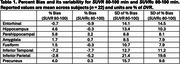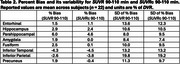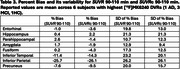# Correcting [^18^F]MK‐6240 *SUVR* for non‐equilibrium effects using tissue clearance correction method does not improve its performance

**DOI:** 10.1002/alz70862_109934

**Published:** 2025-12-23

**Authors:** Praveen Honhar, Jessie Fanglu Fu, Cristina Lois, Arun Garimella, Keith A. Johnson, Julie C Price

**Affiliations:** ^1^ Athinoula A Martinos Center for Biomedical Imaging, Massachusetts General Hospital, Harvard Medical School, Charlestown, MA USA; ^2^ Massachusetts General Hospital, Harvard Medical School, Boston, MA USA; ^3^ Departments of Neurology and Radiology, Massachusetts General Hospital, Harvard Medical School, Boston, MA USA

## Abstract

**Background:**

Honhar et al. (PMCID:10993874) reported a method for bias and noise correction in standardized‐uptake value ratios (*SUVR*, simplified outcome), relative to distribution volume ratios (*DVR*, quantitative gold‐standard), without the need for dynamic data outside *SUVR* time‐window. The method performed well for rapidly‐reversible radiotracers, [^18^F]FE‐PE2I and [^11^C]LSN3172176 (i.e., radiotracers kinetics described by 1‐tissue compartment or simplified reference tissue models). Herein, we tested this method on human [^18^F]MK‐6240 PET data.

**Methods:**

Dynamic [^18^F]MK‐6240 PET data (0‐120 min) in 22 human participants (age: 61±18 y, 8 females, 16 cognitively unimpaired controls [HC], 5 mild cognitive impairment [MCI], 1 Alzheimer’s disease [AD]) were reanalyzed. Metabolite‐corrected arterial input functions were used to compute regional 2‐tissue compartment model *DVR* values (reference: eroded cerebellar gray matter). Regional *SUVR* (80‐100 min and 90‐110 min) values were corrected as: *SUVR*_C_ =*SUVR* / [1‐*β*_ref_/*k_2_*_,ref_+*β*_tar_ *SUVR*/(*R*_1_ *k_2_*_,ref_)], where *SUVR*_C_: corrected *SUVR*, *k_2_*_,ref_: population‐based rate constant for efflux of the radiotracer from reference region, [*β*_ref_,*β*_tar_]: clearance rates of the radiotracer from the reference and target tissues during *SUVR* time‐window, *R*_1_=0.75 (population‐averaged value for ratio of target:reference radiotracer delivery rate). The primary outcome measures were the percent bias (%bias) in *SUVR* (and *SUVR*c) relative to *DVR*, and the standard deviation of %bias.

**Results:**

Across subjects and selected regions of interest (ROIs, Tables 1‐3), the mean %bias was 0.9% for *SUVR* 80‐100 min and 1.2% for *SUVR* 90‐110 min. The variability in %bias was 13.1% for *SUVR* 80‐100 min and 11.9% for *SUVR* 90‐110 min. The mean %bias in *SUVR*c across subjects and ROIs was comparable for *SUVR*c 80‐100 min (‐1.3%) and *SUVR*c 90‐110 min (‐0.1%), with similar variability (∼11%) for *SUVR*c 80‐100 min and *SUVR*c 90‐110 min. Regional measures for (averaged across subjects) are listed in Tables 1‐2. Similar performance metrics were observed for a subcohort analysis of 5 high‐binding individuals with highest average regional *DVR* values (1 AD,3 MCI,1 HC).

**Conclusions:**

[^18^F]MK‐6240 *SUVR* in this cohort was already minimally biased; *SUVR*c did not significantly decrease the variability in *SUVR* bias. This *SUVR* correction method might not be beneficial for radiotracers that deviate considerably from its assumption of 1‐tissue compartment model kinetics.